# Biomimetic Design and Mechanical Properties of Additively Manufactured Titanium Alloy Implant with Gradient Gyroid Structure

**DOI:** 10.3390/ma19173806

**Published:** 2026-09-07

**Authors:** Runze Li, Chenchen Tian, Zikui Wu, Yi Lu

**Affiliations:** 1 School of Mechanical Engineering, Shandong University, Jinan 250061, China; lirunze@mail.sdu.edu.cn (R.L.); zikuiwu@mail.sdu.edu.cn (Z.W.); 2 Key Laboratory of High Efficiency and Clean Mechanical Manufacture of Ministry of Education, Shandong University, Jinan 250061, China; 3 National Key Laboratory of High-End Equipment and Advanced Technology for Metal Forming, Shandong University, Jinan 250061, China; 4 College of Mechanical and Electronic Engineering, Nanjing Forestry University, Nanjing 210037, China; yi_lu@njfu.edu.cn

**Keywords:** bone scaffold, lattice structure, additive manufacturing, titanium alloy, biomimetic

## Abstract

Titanium alloy bone scaffolds have been widely used in the clinical treatment of bone defects. However, conventional titanium alloy bone scaffolds exhibit a lack of porous structure and excessively high elastic modulus, resulting in poor osseointegration. In this study, mimicking the structural characteristics of human bone—namely dense exterior and porous interior—three types of bionic Gyroid titanium alloy bone scaffolds with a uniform porosity of 50% but distinct gradient properties were designed, including uniform lattice structure, linear gradient structure, and quadratic gradient structure. Process optimization of selective laser melting (SLM) and characterization of the as-fabricated microstructures were carried out. The tensile and compressive properties of additively manufactured titanium alloy bone scaffolds were investigated via mechanical testing and finite element analysis (FEA). The results demonstrate that optimized SLM parameters yield a matrix relative density of 98.56% for solid Ti-6Al-4V reference specimens. Using these parameters, bionic gradient-porosity Gyroid bone scaffolds were successfully manufactured. The bionic quadratic function gradient design achieves optimal modulus matching (10–30 GPa) and sufficient mechanical strength at the design level, satisfying the mechanical requirements for bone scaffolds and showing favorable application potential for load-bearing bone scaffolds.

## 1. Introduction

As the fundamental tissue that supports body morphology, ensures motor coordination, and protects vital internal organs, bone maintains structural integrity that is directly critical to normal human physiological functions. Bone defect refers to a common clinical condition characterized by the disruption of bone tissue continuity and integrity, which is mainly caused by various factors including high-energy trauma, chronic infectious diseases, bone tumor resection, debridement of osteomyelitis lesions, and congenital bone dysplasia. Statistically, approximately 4 million people worldwide require bone graft or bone replacement surgery for bone defects each year. Bone grafting, which replaces defective bone tissue and restores its function, has become a standard clinical protocol for bone defect treatment. In terms of clinical usage and market demand, it is second only to blood transfusion, rendering it an indispensable approach in clinical therapy. In particular, the repair of large-segment defects of weight-bearing bones imposes more stringent requirements on the mechanical load-bearing capacity and biocompatibility of bone graft substitutes [1]. Therefore, exploring effective therapeutic strategies for bone defects is of great clinical significance and practical value in improving patients’ quality of life and reducing the incidence of clinical complications.

Bone grafting for clinical bone defects is mainly classified into three categories: autologous bone grafting, allogeneic bone grafting, and artificial bone implantation. Autologous cancellous bone grafting features excellent biocompatibility and is commonly used for small bone defects, yet it is limited by donor-site injury and restricted bone volume. Constrained by the critical threshold of natural bone repair, it also tends to cause adverse outcomes such as bone resorption postoperatively, thereby compromising therapeutic efficacy. Allogeneic bone grafting can resolve the issue of insufficient bone volume, but its clinical application is restricted by low osseointegration efficiency between donor bone and host bone, immune rejection, and other limitations. In recent years, with the interdisciplinary integration of materials science, biomedical engineering, and regenerative medicine, bone graft substitutes (namely artificial bone scaffolds) have emerged, which can be tailored to meet the bone defect requirements of individual patients by precisely regulating material composition, pore structure, and mechanical properties. Evidently, the medical field presents enormous clinical demand and development potential for high-performance, personalized bone scaffolds with both bioactivity and mechanical stability.

Titanium alloys are widely adopted for fabricating bone scaffolds owing to their superior properties, including high strength, low density, non-toxicity, favorable biocompatibility and excellent corrosion resistance. Nevertheless, solid titanium-alloy bone scaffolds possess an excessively high elastic modulus relative to human bone tissue, which readily induces a stress-shielding effect with surrounding tissues and severely impairs the osseointegration capacity of titanium-alloy scaffolds. To address this issue, porous titanium-alloy bone scaffolds have been developed. Relevant studies demonstrate that porous architectures can tune the mechanical properties of scaffolds to match those of bone tissue, thereby mitigating the stress-shielding effect. Meanwhile, such structures create favorable conditions for osteoblast adhesion and proliferation, nutrient transport and bone-tissue ingrowth, further facilitating osseointegration [2]. However, conventional subtractive and formative manufacturing techniques cannot realize high-precision digital fabrication of titanium-alloy bone scaffolds with artificially designed complex pore morphologies, which greatly restricts the structural-design freedom of scaffold pores. Existing reviews have summarized pertinent design considerations for porous Ti-6Al-4V implants and tissue-engineering lattice structures [3].

Advances in additive-manufacturing technology have revitalized the development of porous titanium-alloy bone scaffolds. Boasting unparalleled advantages in the fabrication of complex porous structures and composite materials, additive manufacturing enables the precise forming of porous titanium-alloy bone scaffolds and substantially expands their structural-design freedom, showing promising application prospects for bone-defect-repair therapy [4]. It has been reported that additively manufactured porous titanium-alloy bone scaffolds have achieved preliminary clinical application. Nevertheless, state-of-the-art additively manufactured porous titanium-alloy bone scaffolds fail to achieve genuine personalized customization and optimal physical matching as well as biocompatibility with human tissues. Recent studies concerning the manufacturability of biomedical titanium alloys provide supplementary background for relevant manufacturing workflows [5]. Major additive-manufacturing processes are capable of producing Ti-6Al-4V porous-lattice and TPMS structures; nevertheless, different forming processes exert remarkable influences on forming accuracy, internal-defect distribution and compressive-fracture behavior. Khrapov et al. [6] systematically investigated the process compatibility and forming characteristics of thin-walled TPMS-based porous Ti-6Al-4V structures fabricated by electron-beam melting (EBM). They confirmed that differentiated matching of process parameters directly modulates the wall-thickness uniformity, pore fidelity and surface quality of TPMS structures, which serves as an essential prerequisite for fabricating high-precision biomimetic porous scaffolds. On this basis, Loginov et al. [7] further revealed the deformation law and fracture mechanism of additively manufactured Ti-6Al-4V cellular structures under compressive loading. They illustrated that pore topology, structural continuity and manufacturing defects collectively determine the load-bearing stability and failure modes of porous scaffolds, offering important references for mechanical-property evaluation and structural optimization of lattice structures. For orthopedic load-bearing implants, fatigue performance under cyclic loading represents an indispensable critical indicator apart from static mechanical performance and pore configurations [8].

In terms of mechanical-performance regulation, researchers have carried out extensive investigations on the structural design and mechanical responses of uniform porous bone scaffolds. Zhang et al. [9] fabricated custom diamond-lattice porous Ti-6Al-4V scaffolds via selective laser melting (SLM) and analyzed the relationship between their structures and load-bearing performance. Nelson et al. [10] explored the influence of stress state on the mechanical behavior of 3D-printed porous Ti-6Al-4V scaffolds fabricated by laser powder-bed fusion. Pei et al. [11] performed biomimetic mechanical design and SLM fabrication of titanium bone implants, while Poltue et al. [12] explored TPMS scaffold architectures intended for bone implant applications.

Nevertheless, uniform lattice structures cannot reproduce the spatial gradient features of natural bone. Nune et al. [13] investigated functionally-graded titanium alloy mesh arrays and reported the osteoblastic response. Song et al. [14] constructed radially-gradient Gyroid tantalum scaffolds using linear and quadratic radial-gradient functions to reduce the equivalent elastic modulus while preserving mechanical performance, without constraining a unified overall average porosity.

This study proposes a lattice-structure implant with gradient porosity, which preliminarily replicates the optimal “externally dense and internally porous” configuration of human bone to balance mechanical strength, lightweight performance and physiological functions. Inspired by bone structural features, lattice structures with porosity decreasing from the central axis toward the outer periphery are designed at a fixed overall porosity of 50%. Two gradient models, namely the linearly varying model and the quadratically varying model, are constructed together with the Uniform Distribution Model featuring a constant porosity of 50%. The mechanical properties of these three lattice models with the identical overall porosity of 50% are systematically compared.

## 2. Materials and Methods

### 2.1. Digital Design

Triply periodic minimal surfaces (TPMS) are a class of special surfaces that exhibit periodicity in the *X*, *Y*, and *Z* directions of three-dimensional space, with a zero mean curvature at any surface point. TPMS are generally approximated and defined via implicit function formulation. The governing equation of the Gyroid surface is presented as follows. Previous studies have also investigated the manufacturability and mechanical behaviors of TPMS-based lattices through experimental characterization [15].

*p*(*r*) = sin*X*cos*Y* + sin*Y*cos*Z* + sin*Z*cos*X* = *C*

where r is the unit vector along the three axes in the Cartesian coordinate system: $X=\frac{2\pi x}{T}$, $Y=\frac{2\pi y}{T}$, $Z=\frac{2\pi z}{T}$, where *x*, *y*, *z* are the coordinates in the Cartesian coordinate system, *T* is the period, and *C* is the threshold parameter. The parameter *C* is correlated with the porosity of the porous structure. To design a porous structure with gradient porosity, *C* must be converted from a constant to a variable that varies with spatial coordinates.

By setting *x* and *y* as variables and *z* as a constant, a radially graded porous structure can be designed, for which the expression of *C*(*r*) is given as follows: $C(r)=k_{1}\sqrt{x^{2}+y^{2}}$+$b_{1}$. By setting *z* as a variable and *x*, *y* as constants, an axially graded porous structure can be designed. In this case, the expression of *C*(*r*) is given as follows: $C(r)=k_{2}z+b_{2}$.

All models designed are cylindrical with a radius of 5 mm and a height of 10 mm, and their structures can be defined as follows:

$$\varphi (r)\;=\;\varphi (x,y,z)\;=\;\sin\frac{2\pi x}{T}\cos\frac{2\pi y}{T}+\sin\frac{2\pi y}{T}\cos\frac{2\pi z}{T}+\sin\frac{2\pi z}{T}\cos\frac{2\pi x}{T}\leq C(r)$$

$$x,\;y,\;z\;\epsilon \;R,0\;\leq x^{2}+y^{2}\leq 25,\;0\;\leq z\;\leq \;10$$

**Figure 1:**
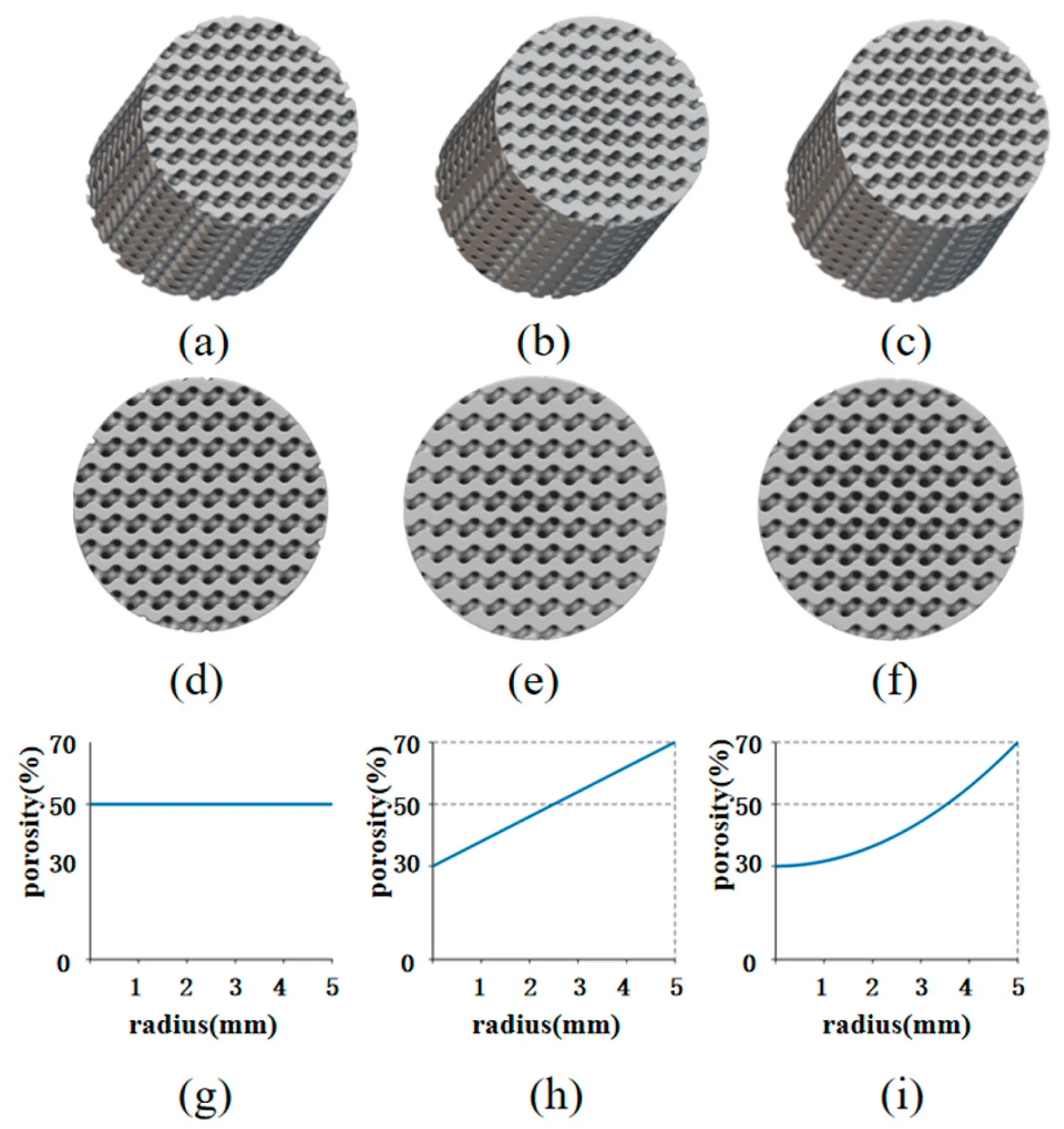
3D models of three types of Gyroid-TPMS scaffold structures. (**a**–**c**) Isometric views of the scaffold assemblies: (**a**) Uniform Distribution Model; (**b**) Linearly Varying Model; (**c**) Quadratically Varying Model. (**d**–**f**) Top-down horizontal cross-section views corresponding to (**a**), (**b**), (**c**), respectively; (**g**–**i**) Porosity-radius curves for each scaffold, illustrating the spatial porosity distribution from central axis to outer boundary. The unit-cell size is 2 mm, and local porosity is adjusted by the threshold parameter C of Gyroid TPMS surfaces.

In this study, nTopology software (version 5.0.4) was used to model three types of lattice structures: the Uniform Distribution Model, the linearly varying model, and the quadratically varying model. Considering the accuracy limitations of SLM additive manufacturing, a unit-cell side length of 2 mm was adopted, which ensures that the minimum pore size achievable during fabrication exceeds 0.5 mm. Figure 1a shows the lattice structure of the Uniform Distribution Model with a uniform porosity of 50%. Figure 1b presents the linearly varying model with an overall porosity of 50%, in which the porosity varies linearly from 30% at the central axis to 70% at the outermost edge following the function. Figure 1c displays the quadratically varying model with the same overall porosity of 50%, where the porosity changes from 30% at the central axis to 70% at the periphery according to the function.

### 2.2. Additive Manufacturing

**Figure 2:**
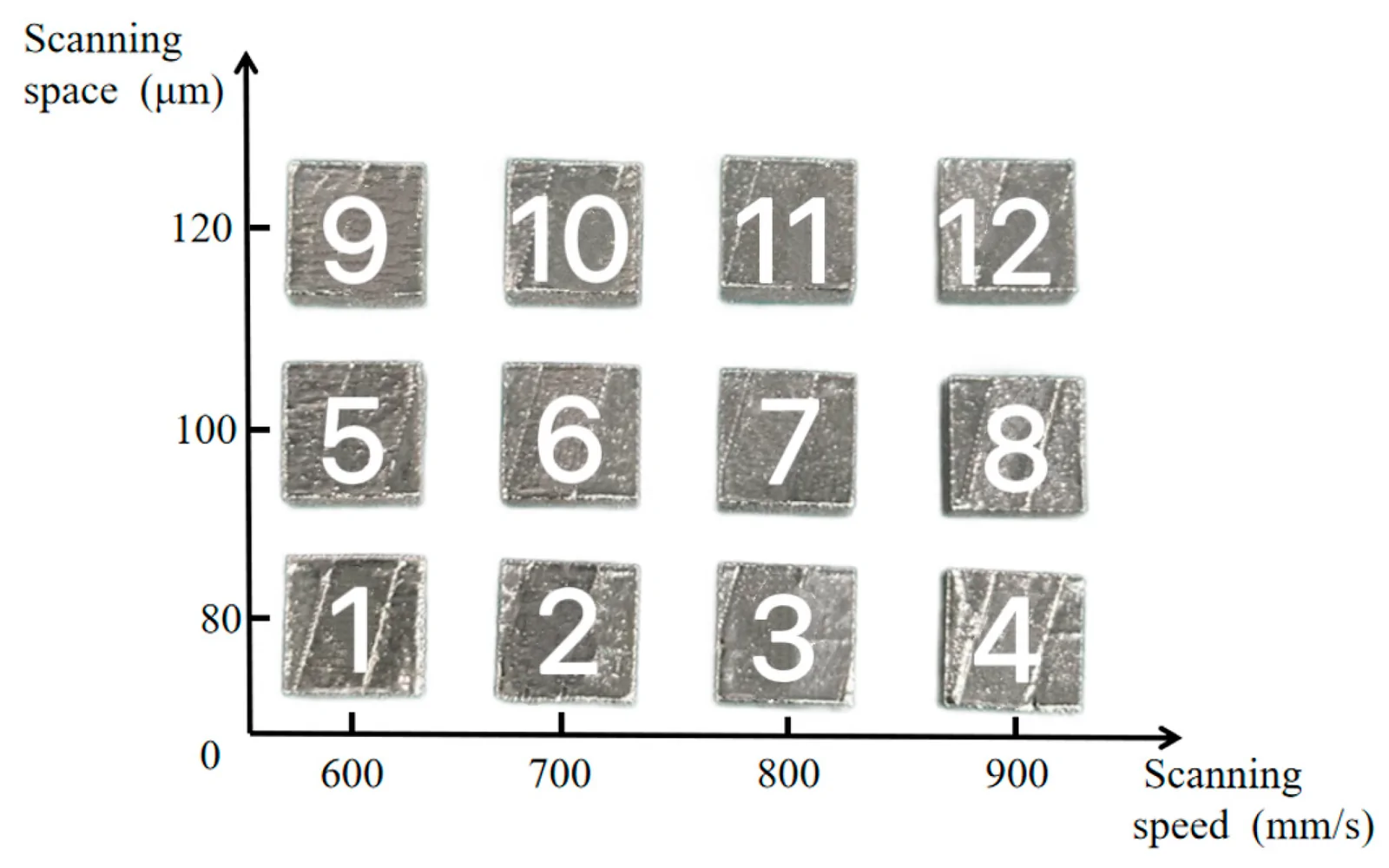
Experimental matrix of process parameters for twelve SLM specimens with varied scanning speed and Scanning space.

In this study, titanium alloy lattice porous structure specimens were fabricated via selective laser melting (SLM). Using a high-energy laser as the heat source, this technique forms three-dimensional components by selectively melting the powder bed layer by layer. Owing to its layer-wise deposition characteristic, SLM enables efficient fabrication of complex porous lattice components. The SLM forming system mainly consists of a laser system, a scanning system, as well as powder feeding, spreading and recovery units. Prior to fabrication, the target lattice structure undergoes 3D modeling and slicing. Inside the build chamber, a powder-spreading mechanism lays titanium alloy powder with a predefined layer thickness. The laser and scanning system work synergistically to selectively melt the powder and realize metallurgical bonding according to the slicing data. After a single layer solidifies, the build platform descends, the powder supply chamber refills powder, and a new powder layer is deposited. The scanning-and-melting procedure is repeated, and the titanium alloy lattice component is finally formed through layer-by-layer accumulation. Unmelted powder can be recycled, and as-built components can satisfy application requirements after post-processing. As listed in Table 1, a full-factorial experimental design was adopted in this work. The laser power was fixed at 190 W, the laser spot diameter was 75 μm, and the powder layer thickness was 30 μm. Three Scanning space (80 μm, 100 μm, 120 μm) and four scanning speeds (600 mm/s, 700 mm/s, 800 mm/s, 900 mm/s) were combined, yielding a total of 12 groups of process parameters. Cubic specimens were fabricated with a GE SLM system under each set of parameters. Figure 2 illustrates the experimental matrix of process parameters for the twelve SLM specimens.

### 2.3. Material Characterization

In this study, the 12 samples were first subjected to density measurement using the Archimedean water displacement method, followed by metallographic characterization including specimen preparation and optical microscopy observation. The Archimedean method was applied to measure the mass of each specimen in air and in a liquid of known density, from which the bulk density of each solid sample was calculated. The relative density was then determined by comparison with the theoretical density of Ti-6Al-4V, allowing quantitative characterization of internal void defects and other metallurgical flaws. The process parameters yielding the optimal relative density were thereby selected.

It should be noted that the relative density measured from solid cubic samples represents the matrix relative density of Ti-6Al-4V material, which reflects the internal consolidation level of the metallic matrix. This value is distinct from the designed macroscopic porosity of Gyroid lattice scaffolds (50%), which originates from intentional open-pore geometric design rather than material-internal void defects.

Metallographic characterization of the additively manufactured specimens was performed as follows: after mounting, grinding, and polishing, the samples were etched using Kroll’s reagent with a volume ratio of V(HF): V(${HNO}_{3}$): V($H_{2}O$) = 1:2:17 for 10–15 s. Finally, optical microscopy was used to observe and analyze the grain morphology, phase composition, and internal defect characteristics, on the basis of which the stability of the forming process was evaluated.

### 2.4. Mechanical Performance Testing

To investigate the influence of different gradient variation modes of porosity on the mechanical properties of lattice structures, quasi-static tensile and compressive tests were carried out on a Zwick/Roell universal testing machine equipped with a calibrated load cell, and the corresponding tensile and compressive stress-strain curves were obtained. The tensile tests were performed following ISO 6892-1 [16], while compressive tests for porous lattice specimens were conducted according to ISO 13314 [17]. Both tensile and compressive tests were conducted at a constant crosshead speed of 1 mm/min at room temperature. The tensile specimen had a length of 20 mm, a width of 6 mm and an overall height of 80 mm, in which the height of the lattice region was 40 mm. The compressive specimen was 20 mm in length, 20 mm in width and 40 mm in height, and the entire specimen consisted of lattice structure. For tensile tests, loading was applied until specimen fracture, while the compressive deformation was set to 10% strain. Strain was derived from the crosshead displacement of the testing machine. Due to the complex porous geometry of lattice specimens, mounting of a contact extensometer was not practical, which may result in systematic deviation in elastic modulus values. The elastic modulus was calculated from the slope of the linear elastic segment of the stress-strain curve. Tensile strength was defined as the maximum stress prior to fracture, and compressive yield strength was determined using the 0.2% offset method. To ensure the reliability of experimental data and eliminate accidental errors, three replicates were tested for each lattice structure under both tension and compression.

### 2.5. Mechanical Performance Simulation

In this study, tensile and compressive mechanical simulations of the lattice structures were conducted using COMSOL Multiphysics (version 6.2). All three models share an identical overall porosity of 50% and thus exhibit consistent volume-averaged equivalent material properties. Accordingly, the same set of standard Ti-6Al-4V equivalent material parameters was assigned to the entire computational domain for all three models. The mechanical distinction among the three models, which is the core focus of this work, originates from their unique internal geometric gradient architectures. Under external loading, these gradient architectures give rise to distinct stress distributions, stress concentration patterns and load-transfer paths, such that the three structures still present distinguishable mechanical behaviors under uniform equivalent material parameters.

The mesh was generated as a physics-controlled mesh consisting of second-order tetrahedral unstructured elements. A mesh convergence analysis was performed via the parametric sweep method, with the bottom reaction force (directly correlated with equivalent stress) selected as the convergence criterion. Comparative calculations were carried out across multiple mesh size levels. The results demonstrate that, for the finally adopted mesh size, the relative deviation of the bottom reaction force is below 5% upon further mesh refinement, which meets the accuracy requirement for macroscopic mechanical property analysis.

The material adopted is annealed Ti-6Al-4V titanium alloy, with a density of 4.5 g/cm^3^, a Young’s modulus of 110 GPa and a Poisson’s ratio of 0.34. The plastic behavior is characterized by the Johnson-Cook constitutive model, with core parameters listed as follows: initial yield strength A = 860 MPa, strain hardening modulus B = 875 MPa, hardening exponent n = 0.386, strain rate sensitivity coefficient C = 0.01, thermal softening exponent m = 0.71, and a reference strain rate of 1 × 10^−4^ s^−1^. All the above parameters are taken from the standard annealed Ti-6Al-4V material library built into COMSOL Multiphysics, which represent widely accepted nominal constitutive parameters for this alloy.

Within the Structural Mechanics module, a linear elastic framework combined with the aforementioned Johnson-Cook plasticity model was employed to accurately capture the nonlinear hardening behavior and strain rate effect of Ti-6Al-4V. Boundary conditions and loading settings were then applied to the model: the bottom surface was defined as a fully fixed constraint, while a tensile or compressive displacement load along the Z-axis was imposed on the top surface. Both tensile and compressive loads were applied through direct displacement constraints on the end faces, with no additional contact pairs configured; the load was transferred uniformly across the bonded end faces. A tensile deformation of 5% and a compressive deformation of 10% were specified respectively, and numerical solutions were obtained using a stationary solver.

After computation, the bottom reaction force was obtained via full-field integration of the Z-direction normal strain. The equivalent stress was calculated as the reaction force divided by the bottom surface area, and the equivalent strain was defined as the ratio of the top displacement to the original height of the model. The equivalent stress–strain curves were thus derived. The simulation model was validated against quasi-static mechanical test results under identical conditions, showing good agreement in overall mechanical trends and key performance parameters. Relevant scaffold experiments, permeability studies, constitutive data and numerical design methods provided references for the modeling work [18,19,20,21].

## 3. Results and Analysis

### 3.1. Selection of Optimal Additive Manufacturing Process Parameters

As shown in Table 2, Sample 8 achieves a density of 4.4351 g/cm^3^, the closest value to that of bulk Ti-6Al-4V titanium alloy (4.5 g/cm^3^) among all 12 samples. Accordingly, the No. 8 parameter group in Table 1—corresponding to Sample 8 in Figure 2—is identified as the optimal SLM process parameters: laser power of 190 W, laser spot diameter of 75 μm, powder layer thickness of 30 μm, scanning space of 100 μm, and scanning speed of 900 mm/s. Tensile and compression specimens were subsequently fabricated using this set of optimal parameters.

Density formula:

$$\rho_{TC4}=\frac{m_{1}}{m_{1}-m_{2}}\rho_{alcohol}$$

### 3.2. Characterization of Porous Morphology

**Figure 3:**
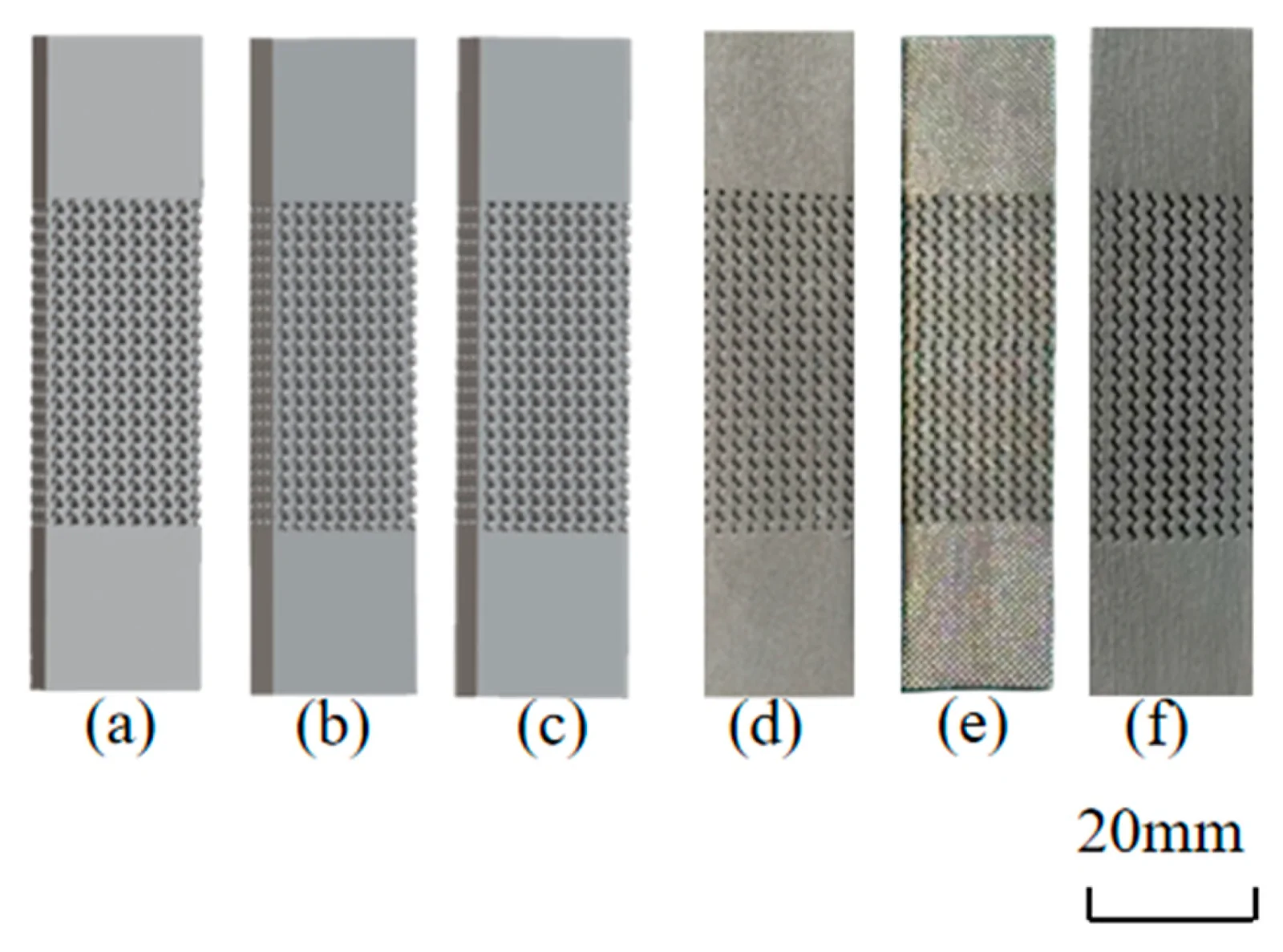
Tensile specimen: (**a**) Uniform Distribution tensile Specimen Model; (**b**) Linearly Varying tensile Specimen Model; (**c**) Quadratically Varying tensile Specimen Model; (**d**) Uniform Distribution tensile Specimen; (**e**) Linearly Varying tensile Specimen; (**f**) Quadratically Varying tensile Specimen. Scale bar: 20 mm.

Figure 3a–c present the design models of the tensile specimens for the three lattice structures, showing the porosity distributions of the uniform lattice structure, linear gradient structure, and quadratic gradient structure, respectively. Figure 3d–f display the morphologies of the additively manufactured tensile specimens corresponding to the three lattice architectures. As illustrated in Figure 3d, the uniform lattice structure exhibits no gradient variation in porosity. In Figure 3e,f, the porosity shows a gradient transition from sparse in the center to dense at the periphery. Notably, the gradient variation is more pronounced in the quadratic gradient structure shown in Figure 3f.

**Figure 4:**
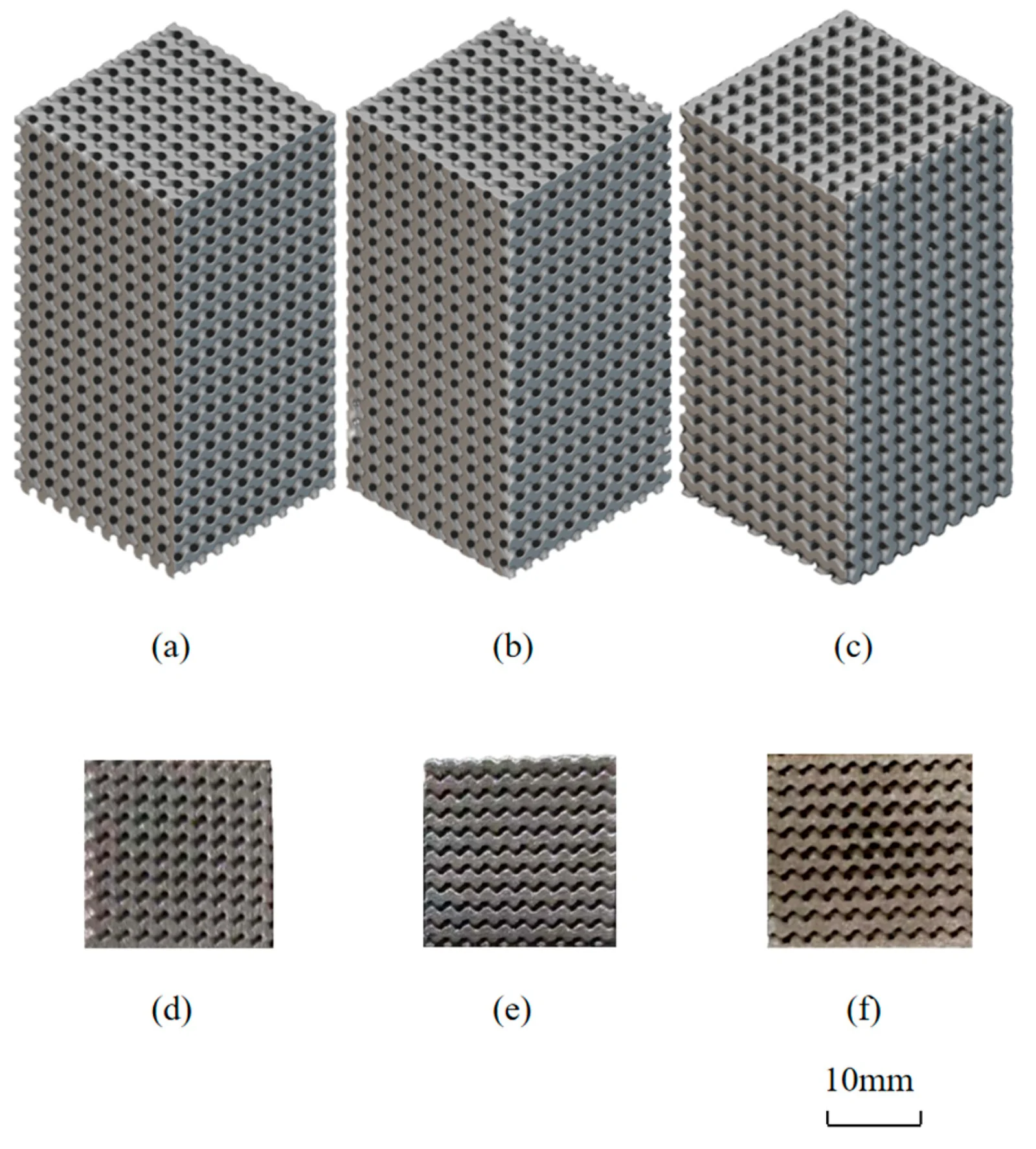
Compression specimens: CAD models of (**a**) uniform distribution, (**b**) linearly varying and (**c**) quadratically varying compression specimens; fabricated samples of (**d**) uniform distribution, (**e**) linearly varying and (**f**) quadratically varying compression specimens. Scale bars: 20 mm (**a**–**c**), 10 mm (**d**–**f**). Scale bar: 10 mm.

Figure 4a–c show the design models of the compressive specimens for the three lattice structures, illustrating the porosity distributions of the uniform, linear gradient, and quadratic gradient structures, respectively. Figure 4d–f display the top-surface morphologies of the additively manufactured compressive specimens corresponding to the three lattice types. As observed in Figure 4d, the uniform lattice structure presents evenly distributed pores without gradient variation. In Figure 4e,f, both the linear and quadratic gradient structures exhibit a gradient porosity feature of “sparse in the center and dense around the periphery”. In particular, the gradient change is more significant in the quadratic gradient structure shown in Figure 4f.

### 3.3. Characterization of Metallographic Structure

**Figure 5:**
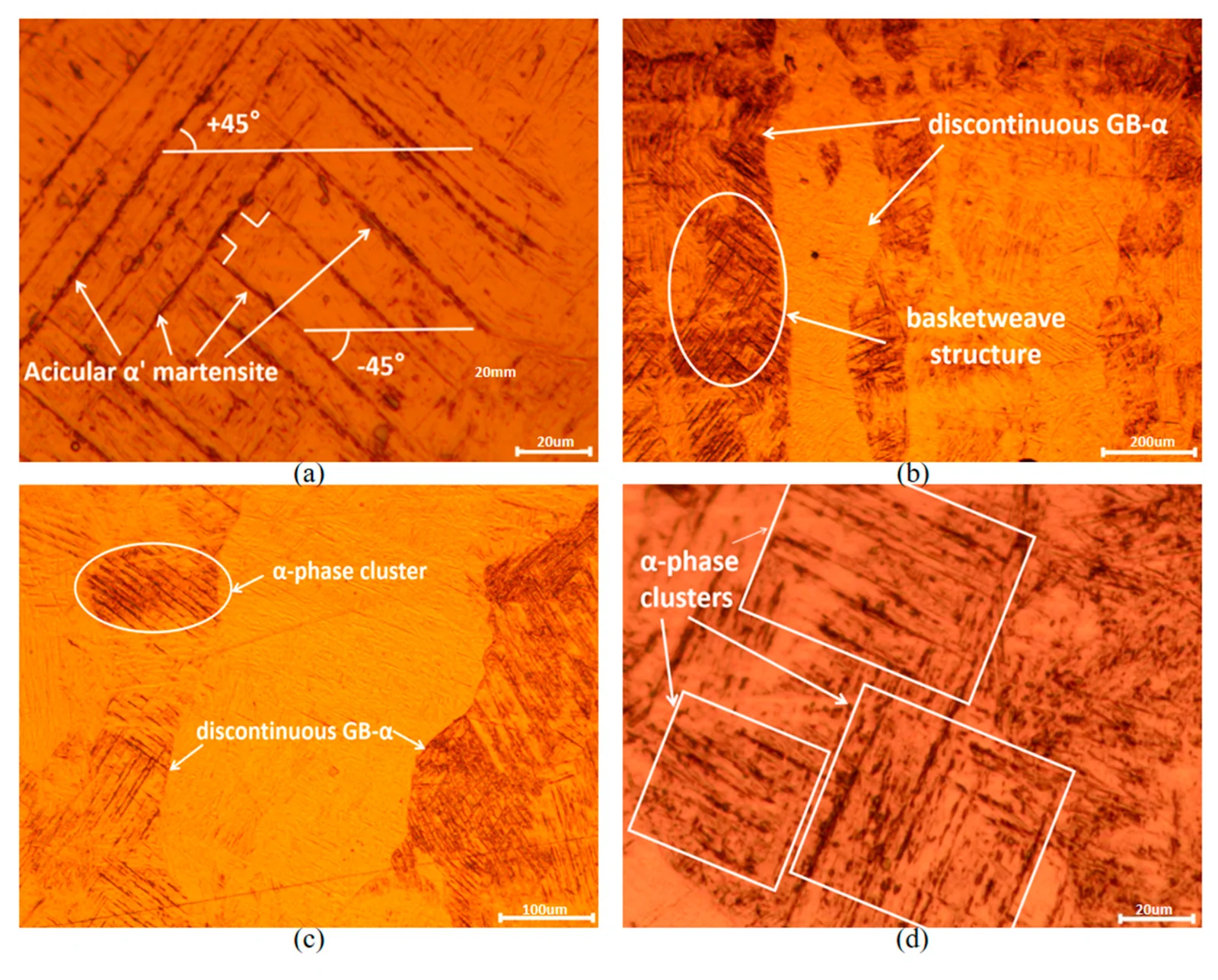
Metallographic microstructure obtained using an optical microscope: (**a**) acicular α′ martensites with ±45° orientation; (**b**) basketweave structure and discontinuous GB-α; (**c**) discontinuous GB-α and α-phase clusters; (**d**) detailed view of α-phase clusters.

The typical metallographic structure of conventional annealed Ti-6Al-4V titanium alloy consists of equiaxed α-phase + transformed β-phase (lamellar α-phase + residual β-phase) [22]. Compared with traditional processing methods, the transient high-temperature melting and ultra-high cooling rate involved in selective laser melting (SLM) induce significant microstructural evolution in Ti-6Al-4V titanium alloy, resulting in a large number of acicular martensites with high aspect ratio, as shown in Figure 5a [23]. In addition, Figure 5a reveals that most acicular martensites grow at an angle of approximately ±45° relative to the SLM building direction. This orientational feature enables effective resistance against compressive stress applied horizontally or vertically to the scanning direction along the 45° planes. Figure 5b,c show the presence of discontinuous grain boundary α (GB-α). A basket-weave microstructure is observed in Figure 5b, which originates from the suppression of diffusive phase transformations due to the ultra-high cooling rate during forming, promoting the formation of acicular martensite via a diffusionless shear mechanism [23]. Comprehensive analysis of Figure 5 indicates that the relatively high scanning speed in SLM is the key processing parameter governing microstructural evolution [24]. During the nucleation and growth of acicular martensite, rapid cooling introduces a high density of dislocation defects inside the crystal lattice. Meanwhile, the amount of acicular α-phase increases and coarsens, forming clustered structures as seen in Figure 5c,d. These features eventually evolve into supersaturated, non-equilibrium acicular martensite, corresponding to a typical diffusionless phase transformation of the β-phase (martensitic transformation) [23].

### 3.4. Comprehensive Mechanical Performance of Gradient Lattice Structure

#### 3.4.1. Tensile Mechanical Performance of Gradient Lattice Structure

**Figure 6:**
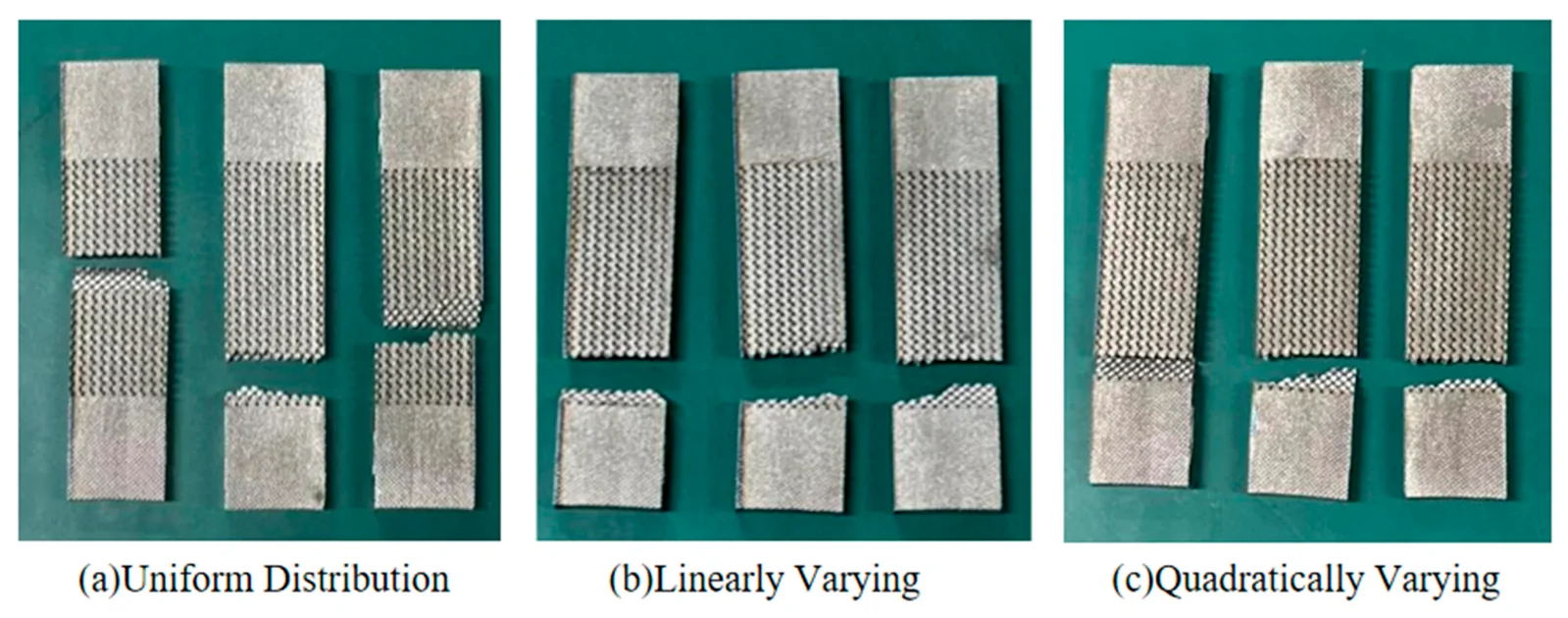
Tensile fractured specimen.

**Figure 7:**
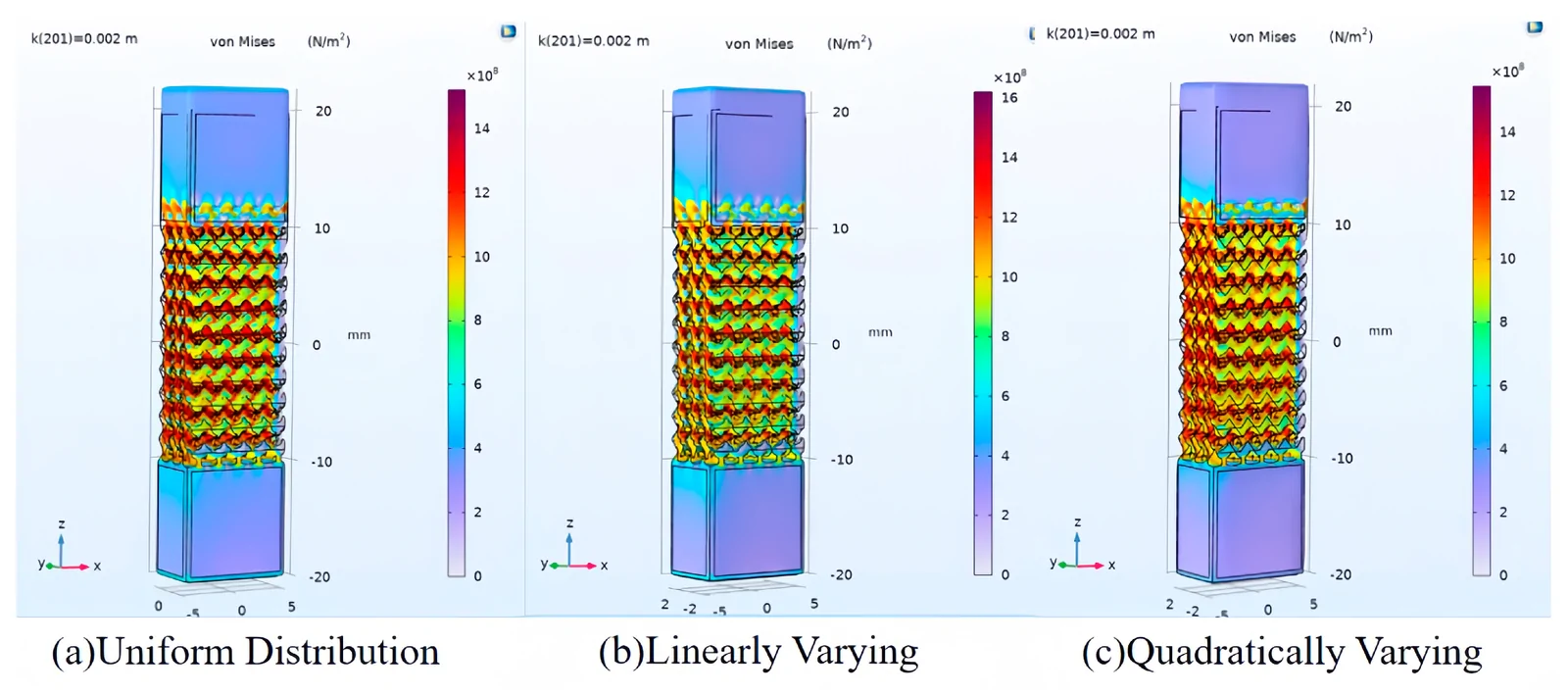
(**a**–**c**) Tensile simulation stress contour plot.

Based on tensile experiments and simulation analysis, the tensile mechanical properties of the three gradient lattice structures were comprehensively compared. Representative fracture morphologies after tensile testing are shown in Figure 6. The von Mises stress distributions obtained from finite-element tensile simulation are presented in Figure 7. After data processing of the results obtained from tensile experiments and finite-element simulations, the corresponding tensile stress-strain curves were acquired.

**Figure 8:**
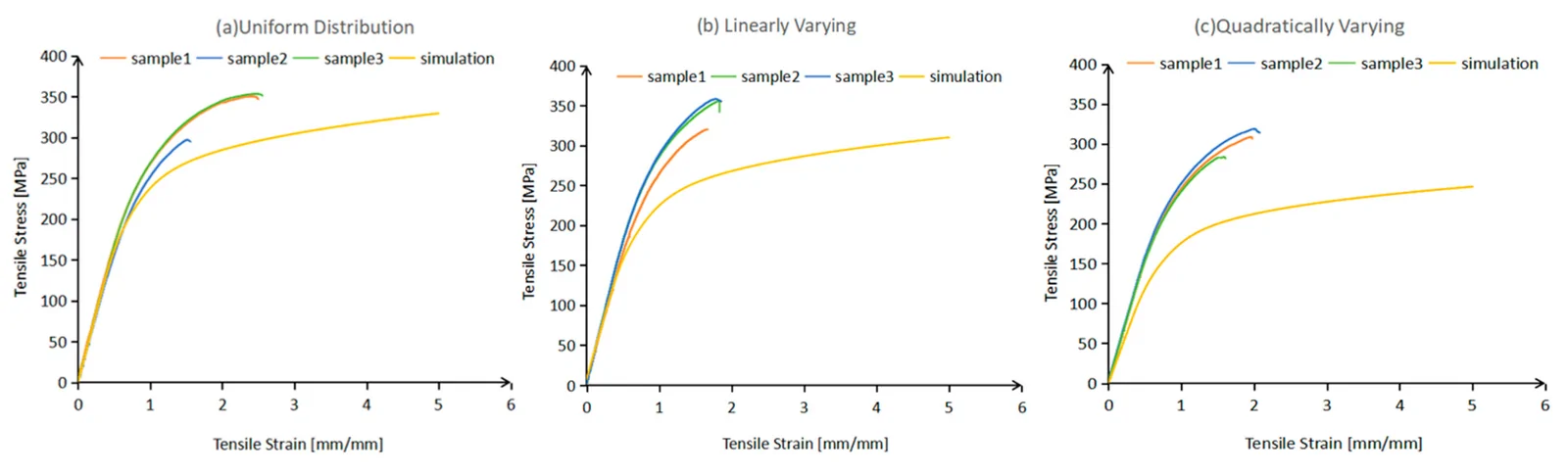
The tensile stress-strain curves from experiments and simulations: (**a**) Uniform Distribution Model; (**b**) Linearly Varying Model; (**c**) Quadratically Varying Model.

As shown in Figure 8, the tensile equivalent stress–strain curves of the three gradient lattice structures intuitively reflect their fundamental tensile mechanical properties. Owing to various factors including experimental errors, model simplification, and boundary conditions, certain deviations exist between the experimental and simulated tensile curves for all three structures, while the overall variation trends remain consistent.

**Figure 9:**
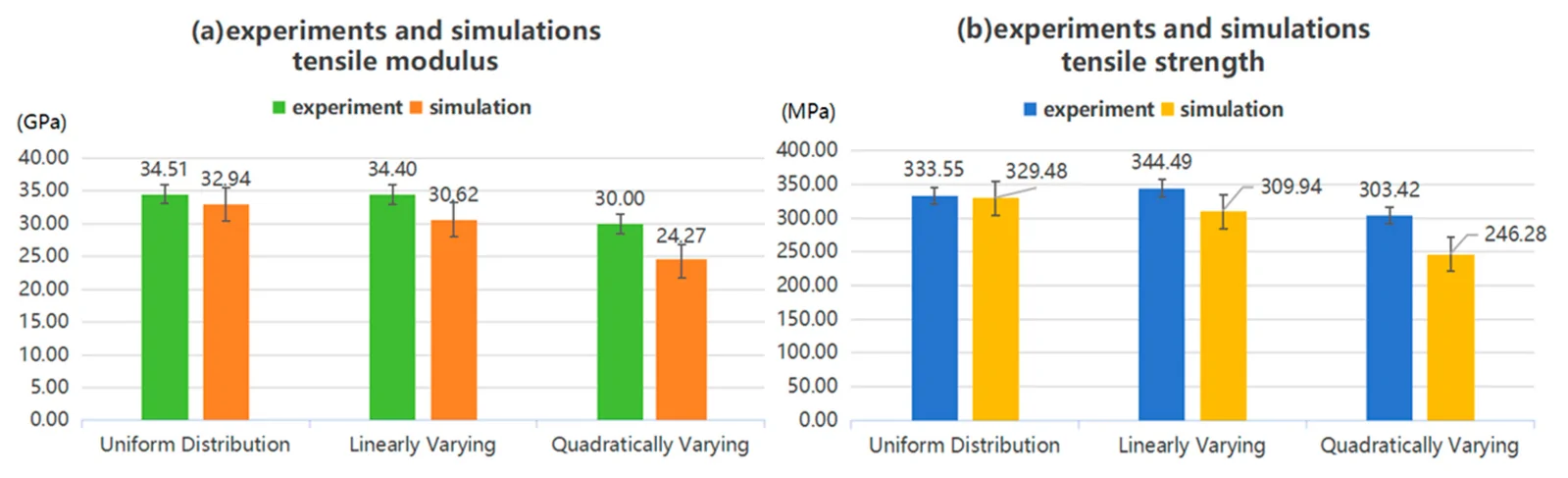
(**a**) Tensile modulus from experiments and simulations; (**b**) Tensile strength from experiments and simulations.

The experimental results show that:For tensile modulus, the experimental values of the uniform lattice structure, linear gradient structure, and quadratic gradient structure are 34.51 GPa, 34.40 GPa, and 30.00 GPa, respectively. The tensile modulus of the uniform lattice structure is comparable to that of the linear gradient structure, while the quadratic gradient structure exhibits the lowest tensile modulus. For tensile strength, the experimental values are 333.55 MPa, 344.49 MPa, and 303.42 MPa, respectively. The linear gradient structure possesses a slightly higher tensile strength than the uniform lattice structure, whereas the quadratic gradient structure shows the lowest tensile strength. Key tensile parameters including modulus and strength from experiments and simulations are further summarized in Figure 9.

The simulation results indicate that: For tensile modulus, the simulated values are 32.94 GPa, 30.62 GPa, and 24.27 GPa, respectively, all of which are slightly lower than the corresponding experimental values. For tensile strength, the simulated values are 329.48 MPa, 309.94 MPa, and 246.28 MPa, respectively, which are also slightly lower than the corresponding experimental values.

The experimental-simulation differences in tensile modulus for the Uniform Distribution Model, linearly varying model and quadratically varying model are 4.55%, 10.99% and 19.10%, respectively, and the differences in tensile strength are 1.22%, 10.03% and 18.84%, respectively. Apart from model simplification and manufacturing defects, the reduction in the number of lattice units in the simulation constitutes a key limiting factor. The full-scale lattice model contains a large number of units. Even with the minimum acceptable surface tolerance, geometric export suffers from excessive computational time and export failure. If geometric fidelity is reduced to complete export, lattice geometric distortion will be introduced and further larger deviations will occur. Therefore, the geometric model was appropriately simplified in this study. The number of lattice units was reduced while the key geometric features of unit-cells were retained to achieve valid simulation.

#### 3.4.2. Compressive Mechanical Performance of Gradient Lattice Structure

**Figure 10:**
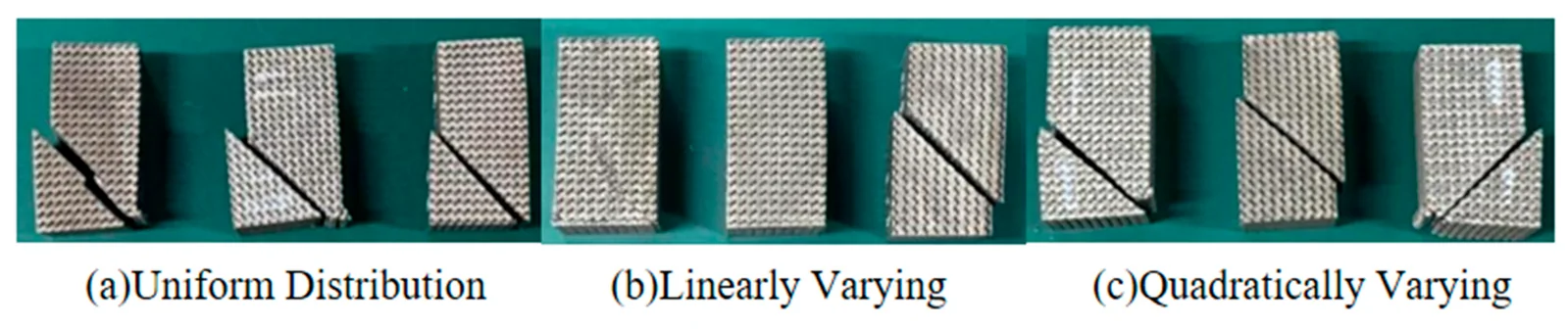
Compressive fractured specimen.

**Figure 11:**
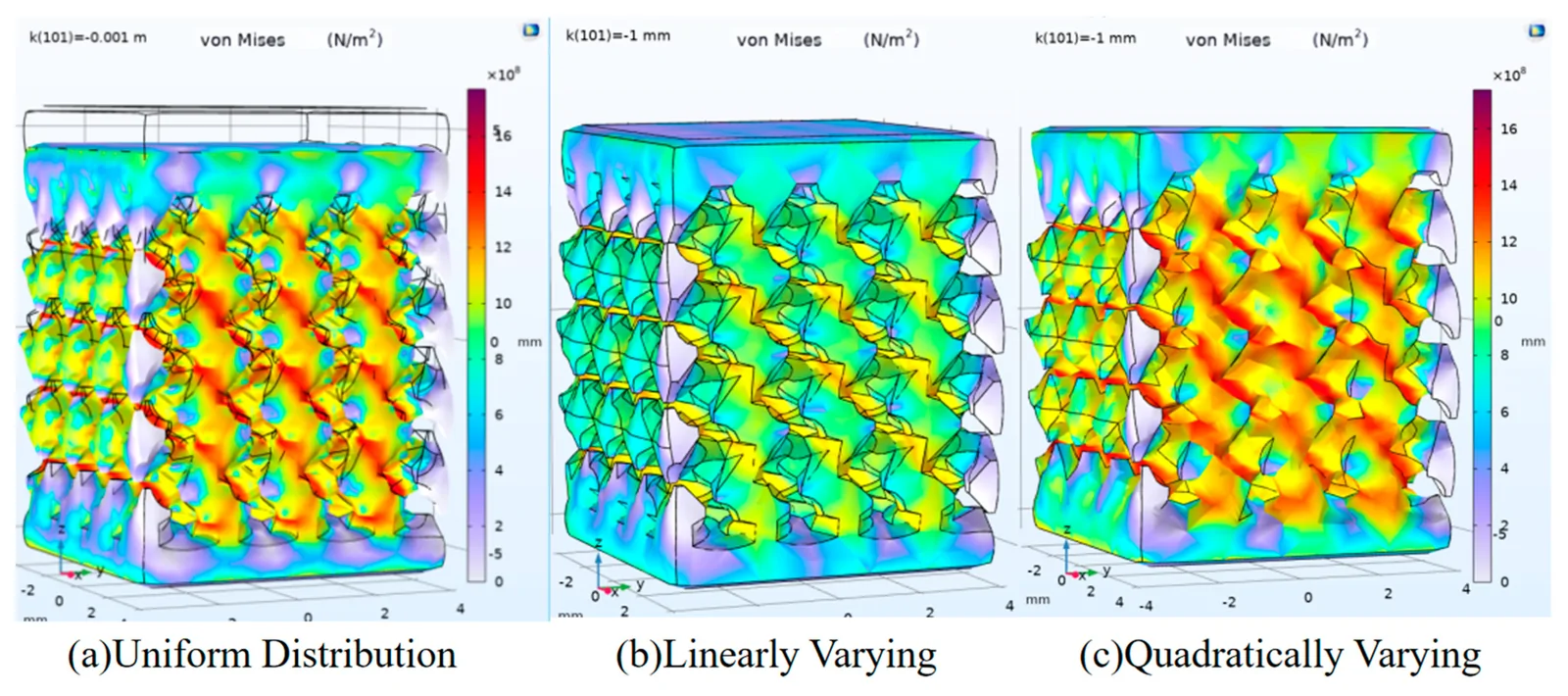
(**a**–**c**) Compressive simulation stress contour plot.

The compressive mechanical properties of the three gradient lattice structures were comprehensively compared based on compression experiments and numerical simulations. The macroscopic fracture features of specimens under compressive loading are provided in Figure 10, while the simulated von Mises stress contours under compression are displayed in Figure 11. After data processing of the results obtained from compression experiments and finite-element simulations, the corresponding compressive stress-strain curves were acquired.

**Figure 12:**
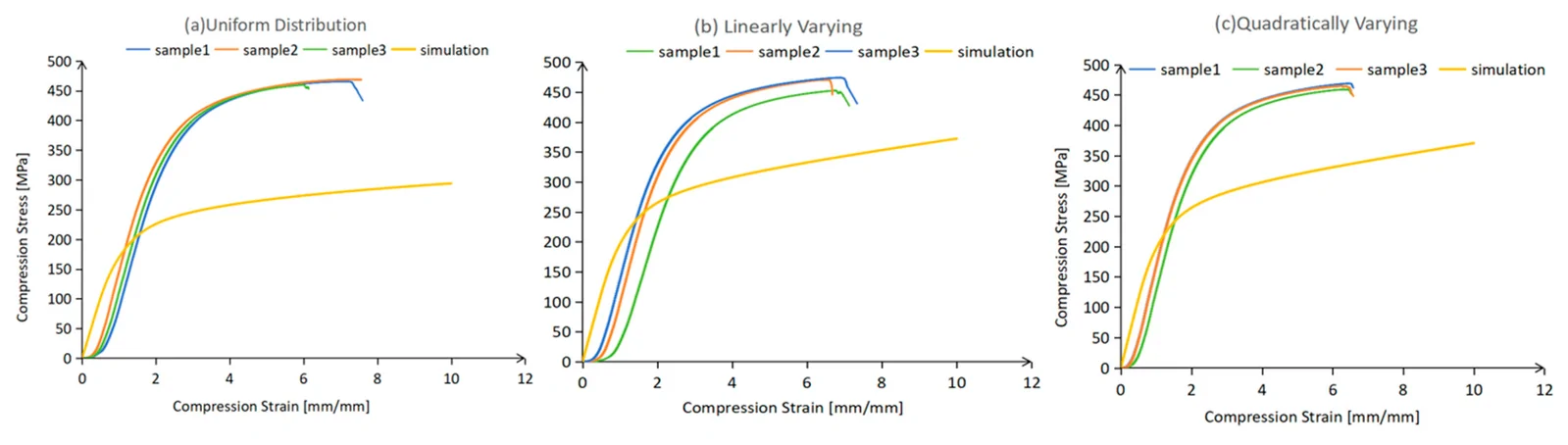
The compressive stress-strain curves from experiments and simulations: (**a**) Uniform Distribution Model; (**b**) Linearly Varying Model; (**c**) Quadratically Varying Model.

As shown in Figure 12, the compressive equivalent stress–strain curves of the three gradient lattice structures intuitively reflect their fundamental compressive mechanical properties. Affected by various factors including experimental errors, model simplification, and boundary conditions, certain deviations exist between the experimental and simulated compressive curves for all three structures, whereas the overall variation trends remain consistent.

**Figure 13:**
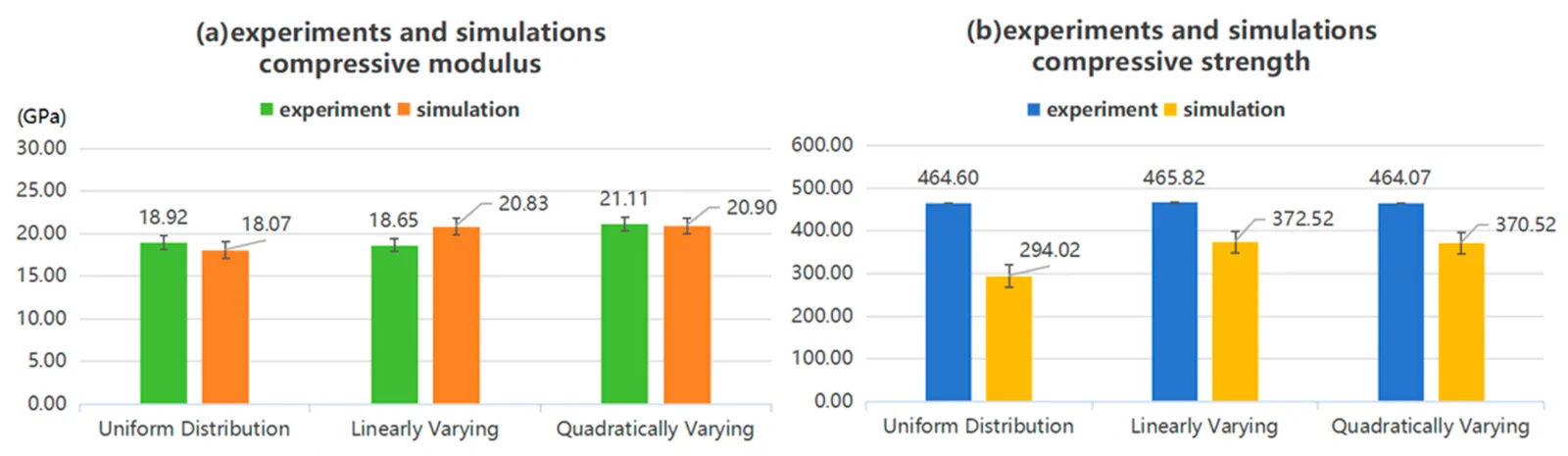
(**a**) Compressive modulus from experiments and simulations; (**b**) Compressive strength from experiments and simulations.

The experimental results show that: For compressive modulus, the experimental values of the uniform lattice structure, linear gradient structure, and quadratic gradient structure are 18.92 GPa, 18.65 GPa, and 21.11 GPa, respectively. The quadratic gradient structure exhibits the highest compressive modulus, while the compressive modulus of the uniform lattice structure is comparable to that of the linear gradient structure. For compressive strength, the experimental values of the uniform lattice structure, linear gradient structure, and quadratic gradient structure are 464.60 MPa, 465.82 MPa, and 464.07 MPa, respectively, with very close values to each other. Key compressive parameters including modulus and strength from experiments and simulations are further summarized in Figure 13.

The simulation results indicate that: For compressive modulus, the simulated values of the uniform lattice structure, linear gradient structure, and quadratic gradient structure are 18.07 GPa, 20.83 GPa, and 20.90 GPa, respectively. The simulated values are relatively close to the experimental values, among which the quadratic gradient structure has the highest compressive modulus. For compressive strength, the simulated values of the uniform lattice structure, linear gradient structure, and quadratic gradient structure are 294.02 MPa, 372.52 MPa, and 370.52 MPa, respectively, all of which are much lower than the experimental values. The compressive strength of the linear gradient structure is comparable to that of the quadratic gradient structure, and both are significantly higher than that of the uniform lattice structure.

The simulation-experimental differences in compressive modulus for the Uniform Distribution Model, linearly varying model and quadratically varying model are 4.49%, 11.69% and 0.99%, respectively, and the differences in compressive strength are 36.71%, 20.04% and 20.16%, respectively. The Uniform Distribution Model exhibits the largest deviation in compressive strength. The comparison shows that the prediction accuracy of elastic modulus is generally higher, while the simulation deviation of compressive strength is more remarkable. Apart from model simplification and manufacturing defects, the main reason lies in the significantly larger number of original lattice units in compressive specimens compared with tensile specimens. Under the same model simplification treatment, the compression condition is more severely affected by unit reduction, leading to greater simulation deviation. Elastic modulus represents a global mechanical response and is slightly affected by local unit removal, which yields good agreement between simulation and experiment. By contrast, strength is highly dependent on local stress states, and the prediction error of compressive strength is further amplified.

#### 3.4.3. Application of Gradient Lattice Structures in Bone Scaffolds Based on Mechanical Performance

Current porous bone-scaffold designs are mainly based on uniform lattices and low-modulus gradient lattice structures. When applied to load-bearing bone repair scenarios, they still exhibit limitations in the precise regulation of mechanical properties, and can hardly satisfy the dual requirements of bearing strength and bone modulus matching simultaneously. Accordingly, drawing on the structural feature of human bone “dense outside and sparse inside”, this study proposes a design scheme for Gyroid bionic implants with gradient porosity. Three types of lattice structures, namely the Uniform Distribution Model, linearly varying model and quadratically varying model, are constructed, and their mechanical response behaviors are systematically compared under a constant overall porosity of 50%.

The experimental results reveal that under the consistent overall porosity of 50%, the measured tensile modulus and compressive modulus of the quadratically varying model are 30.00 GPa and 21.11 GPa, respectively, both falling within the human bone elastic-modulus range (10–30 GPa) referenced in this study [25]. It is the only configuration among the three structures that satisfies the modulus-matching principle. This feature is expected to mitigate the stress shielding effect after implantation and achieve better mechanical compatibility with human load-bearing bone. In terms of bearing strength, the tensile and compressive strengths of the three structures are all higher than the reference load level of load-bearing sites such as the femur. Among them, the strength of the quadratically varying model is comparable to the magnitude of cortical bone, meeting the mechanical prerequisites for load-bearing bone applications [26].

Overall, the quadratically varying model realizes the synergistic regulation of scaffold modulus and strength through gradient variation of porosity. It presents prominent advantages in optimizing the mechanical performance of bone scaffolds and can provide references for the structural design of potential implants for load-bearing bone defect repair.

## 4. Discussion

Recent studies have focused on the topology, manufacturing fidelity and mechanical responses of porous titanium scaffolds. Li et al. [27] reviewed the preparation and post-processing methods for 3D-printed porous titanium alloys, while Wang et al. [28] investigated Spinodoid bone scaffolds using a data-driven inverse design approach. Other studies have examined the manufacturing and compressive behaviors of TPMS structures [6,7]. The measured compressive modulus of the quadratically varying model Gyroid structure in this work is 21.11 GPa. Nevertheless, comparisons across different studies should be treated with caution due to differences in topology, porosity, materials, processing routes and test protocols.

This study has four main limitations. First, the characterization of as-built structures and processes is insufficient. Quantitative micro-CT characterization was not performed on the fabricated gradient scaffolds, so the actual porosity, pore-size distribution, strut wall thickness and dimensional deviations from the designed model cannot be quantified. The relative density of the matrix was obtained only from solid specimens, and manufacturing defects at the strut level of lattices were not evaluated. Moreover, no parallel specimens were prepared for each group during the preliminary selection of SLM process parameters. Parameters were preliminarily screened merely based on matrix relative density and metallographic observations, lacking rigorous process verification with multiple specimens and multi-index evaluation. Second, the coverage of mechanical tests is limited. Only quasi-static tensile and compressive tests were carried out, and the fatigue performance under cyclic loading, which is critical for load-bearing implants, was not evaluated [26]. Each group contained only three replicate specimens with a limited sample size, and statistical significance analysis between groups was not conducted. Third, simplifications exist in the numerical model. Geometric simplification was adopted in finite-element simulations, which limits the accuracy of local stress distribution. The simulation can well reflect the overall trend of stiffness variation, but obvious deviations occur in strength prediction. Fourth, insufficient biomechanical and biological validations are available. The modulus-matching analysis in this study is based on a general reference range. The actual in-vivo biomechanical performance and stress-shielding effect still require further verification combined with specific anatomical conditions and implant-bone interface behaviors. Meanwhile, XRD phase analysis, in-vitro cell culture and in-vivo animal experiments were not performed. The results of this study only demonstrate that gradient design can regulate the mechanical properties of scaffolds. They cannot confirm tissue ingrowth, osteogenic effect, osseointegration performance or clinical applicability. Conclusions regarding tissue ingrowth, osteogenesis, osseointegration and definite phase identification are beyond the scope verified in the present work. Future work will incorporate calibrated as-built geometric data, XRD phase analysis, fatigue tests and biological validations to further clarify the application potential of the proposed structure.

## 5. Conclusions

Based on bionic design and additive manufacturing technology, this study designed and prepared three types of lattice structures for bone scaffolds, including uniform lattice structure, linear gradient structure, and quadratic gradient structure. The mechanical properties of the three bone scaffold lattice structures were investigated through experiments and simulation techniques. The main research conclusions are as follows:

1. Selective Laser Melting (SLM) has significant advantages in the preparation of titanium alloy bone scaffolds, which can realize the good forming of gradient lattice structures. Through the additive manufacturing process experiment, the optimal process parameters suitable for Ti-6Al-4V titanium alloy bone scaffolds were obtained: laser power of 190 W, laser spot diameter of 75 μm, powder layer thickness of 30 μm, scanning space of 100 μm, and scanning speed of 900 mm/s. Under these conditions, the solid Ti-6Al-4V reference sample achieves a matrix density of 4.4351 g/cm^3^ (matrix relative density of 98.56%). These optimized parameters were subsequently adopted for manufacturing gradient Gyroid lattice specimens.
2. Comprehensive analysis of tensile and compressive mechanical properties shows that the experimental and simulation results of the three gradient lattice structures maintain good consistency in the trend of stiffness. In terms of tensile properties, the linear gradient structure has the highest strength, while the quadratic gradient structure has the lowest strength. In terms of compressive properties, the quadratic gradient structure has the highest modulus, and the uniform lattice structure and linear gradient structure have similar moduli. The simulation can accurately predict the stiffness change law, but the predicted strength is generally lower. The consistency of the tensile law is higher, while there is a certain difference in the compressive strength law. This is mainly because compressive specimens have more lattice units, requiring greater unit deletion under identical simplification and causing larger deviations. Elastic modulus, as a global response, matches experiments well, while strength governed by local stresses yields higher compressive prediction errors.
3. Among the three designs, the quadratically varying model exhibits the optimal combination of measured modulus and strength. At an overall porosity of 50%, its tensile modulus is 30 GPa and compressive modulus is 21.11 GPa, which fall within the human bone comparison range adopted in this study [25]. Its strength also exceeds the nominal load level used for design comparison. The quadratically varying model regulates mechanical properties through gradient variation of porosity, and its strength level is comparable to that of cortical bone, satisfying the mechanical prerequisites for load-bearing bone applications [26]. These results only support its mechanical feasibility; tissue ingrowth, osteogenesis and clinical performance still need to be verified via In-Vitro and In-Vivo experiments.

## Figures and Tables

**Table 1:** Laser parameter optimization.

Manufacturing Parameter	Laser Power (W)	Spot Diameter (μm)	Layer Thickness (μm)	Scanning Space (μm)	Scanning Speed (mm/s)
1	190	75	30	80	600
2	190	75	30	80	700
3	190	75	30	80	800
4	190	75	30	80	900
5	190	75	30	100	600
6	190	75	30	100	700
7	190	75	30	100	800
8	190	75	30	100	900
9	190	75	30	120	600
10	190	75	30	120	700
11	190	75	30	120	800
12	190	75	30	120	900

**Table 2:** Density results of additively manufactured Ti-6Al-4V titanium alloy.

Number	m_1_	m_2_	Ρ/g/cm^3^
1	2.3998	1.9589	4.2945
2	2.4350	1.9978	4.3944
3	2.4399	2.0034	4.4103
4	2.4103	1.9806	4.4257
5	2.4646	2.0226	4.3995
6	2.4540	2.0162	4.4226
7	2.4098	1.9805	4.4289
8	2.3980	1.9714	4.4351
9	2.4248	1.9895	4.3951
10	2.3876	1.9621	4.4273
11	2.3495	1.9314	4.4338
12	2.3650	1.9439	4.4312

## Data Availability

The original contributions presented in this study are included in the article. Further inquiries can be directed to the corresponding author.
